# Prevalence of malnutrition and associated factors among under-five children in Ethiopia: evidence from the 2016 Ethiopia Demographic and Health Survey

**DOI:** 10.1186/s13104-019-4444-4

**Published:** 2019-07-11

**Authors:** Abay Kassa Tekile, Ashenafi Abate Woya, Garoma Wakjira Basha

**Affiliations:** Statistics Department, Science College, Bahir Dar University, P.O. Box 79, Bahir Dar, Ethiopia

**Keywords:** Under-five children, Underweight, Wasting, Stunting, Ethiopia

## Abstract

**Objective:**

The aim of this study was to assess the risk factors for malnutrition among children aged 0–59 months in Ethiopia. The analyzed data were obtained from the 2016 EDHS and 9495 under-5 years’ children were considered in this analysis. The data was extracted, edited and analyzed by using SPSS Version 23.0. Both bivariate and multivariable binary logistic regression model was used to identify the determinants of children malnutrition.

**Results:**

The prevalence of stunting, wasting, and underweight were 38.3%, 10.1%, and 23.3%, respectively. About 19.47% of children were both stunted and underweighted, and only 3.87% of children had all the three conditions. Among the factors that considered in this study, age of a child, residence region, mothers’ education level, mothers’ BMI, household wealth index, sex of a child, family size, water and toilet facility were significantly associated with malnutrition in Ethiopia. The authors concluded that malnutrition among under-five children was one of the public health problems in Ethiopia. Therefore, the influence of these factors should be considered to develop strategies for reducing malnutrition in Ethiopia.

**Electronic supplementary material:**

The online version of this article (10.1186/s13104-019-4444-4) contains supplementary material, which is available to authorized users.

## Introduction

Malnutrition among under-5 year children is a common public health problem and it is one of the main reasons for the death of children in developing countries [1]. As of the World Health Organization report, about 35% of under-five children’s death is associated with malnutrition in the world [2]. There are 165 million stunted, 99 million under-weighted, and 51 million wasted children globally [3].

The prevalence of stunting has decreased from 58% in 2000 to 44% in 2011 in Ethiopia. The prevalence of wasting is changed from 12% in 2000 to 10% in 2011. The prevalence of underweight has consistently decreased from 41% in 2000 to 29% in 2011 [4]. In Tanzania, a high prevalence of underweight (46.0%), stunting (41.9%) and wasting (24.7%) are observed in 2017. In addition, 33% of children are both stunted and underweight, 21% of children are underweight and wasted, and 12% of children are stunted and wasted [5]. In Ethiopia, more than one-third of a child deaths are associated with malnutrition [6]. Moreover, the proportion of malnutrition is higher among anemic children compared to those of non-anemic [7].

Different researchers conducted a study on malnutrition among under-five children in different parts of the country. These studies were mainly focused on the prevalence and determinants of malnutrition among under-five children, but they gave little attention for exploring the relationship between under-five malnutrition and child anemia. So, the main aim of this study was to explore the major factors of malnutrition and its association with anemia by using updated data from the 2016 EDHS.

## Main text

### Study design and sampling

The 2016 Ethiopia Demographic and Health Survey data was used for this study. The 2016 EDHS used a two-stage stratified sampling design to select households. In the first stage, there were 645 enumeration areas (202 in urban and 443 in rural areas) based on the 2007 Ethiopia Population and Housing Census (PHC). A total of 18,008 households were considered, of which 16,650 (98% of response rate) households were eligible. The women were interviewed by distributing questio-ners and information on their birth history and 9495 under-five children were considered for this study [8].

### Measurements

The dependent variable for this study was the malnutrition status of under-5 year children (stunting, underweight and wasting). Children whose height-for-age Z-score is below minus two standard deviations (− 2 SD) from the median of the reference population are considered as stunted. If the weight-for-age Z-score is below minus two standard deviations (− 2 SD) from the median of the reference population then the child is underweight. Children whose weight for height Z-score is below minus two standard deviations (− 2 SD) from the median of the reference population are considered as wasted [8]. Socio-demographic, socio-economic and health-related variables were considered as independent variables in this study.

### Statistical data analysis

The data were extracted, edited, and analyzed by using SPSS version 23 for Windows. Then a weighted analysis was conducted using the same sampling weight given for each region in Ethiopia DHS to compensate for the unequal probability of selection between the strata [8, 12]. Bivariate logistic regression was performed and variable with P-value less than 0.25 were transported into multivariable binary logistic regression analysis to identify the determinant of malnutrition of under-five children. Finally, variables with P-values < 0.05 in the multivariable logistic regression model were taken as statistically significant.

### Results

Samples of 9495 under-five children were considered in this research. The weighted prevalence of stunting, underweight, and wasting were 38.3%, 23.3%, and 10.1%, respectively. About 66% of interviewed mothers had no education and only 2% of them attended higher education. About 44% of children were found between 0 to 24 months and more than half (51.1%) were males. Only 11% of the respondents were from urban areas and 32% were in the rich wealth index. Around 20% of children’s mother were underweighted (having body mass index less than 18.5) (Additional file 1).

#### Determinants of stunting

Among the factors that considered in this study, child’s age, residence region, mothers’ education level, wealth index, child sex, toilet facility, size of a child, mothers’ BMI and number of children per household were associated with stunting. Compared to children of 0–24 months, the odds of stunting among children in the age group of 25–47 months were 2.645 times higher. The child in the age group of 48–59 months was 1.763 times higher. Compared with children in Tigray region, the risk of being stunted was decreased by 32%, 33%, and 60%, among children living in Afar, Oromia, and Somali regions, respectively. The risk of being stunted among children whose mothers attended primary education was 0.87 times less compared to children whose mother did not attend education. The risk of being stunted among children whose mothers attended secondary and higher education were 0.606 and 0.453 times less compared to children whose mother did not attend education respectively. Compared to male children, the probability of being stunted among female children was decreased by 16%. Compared to children living in households with poor economic status, the odds of being stunted among children living in households with medium and rich economic status were decreased by 20% and 31%, respectively.

Children born with small size were 1.509 times more likely to be stunted than children born larger (AOR = 1.509; 95% CL 1.332–1.709) and children who had born with medium size were 1.189 times more likely to be stunted than children born larger (AOR = 1.062; 95% CL 1.062–1.331). Children born to underweight mothers (BMI < 18.5) were 2.163 (AOR: 2.163, 95% CI 1.750, 2.673) times more likely to be stunted compared to those born to overweight mothers (Table 1).

**Table 1:** Determinants associated to stunting (EDHS, 2016)

Variables	P-value	COR (95% CI)	P-value	AOR (95% CI)
Age of child in months				
0–24 (ref)	< 0.0001	1	< 0.0001	1
25–47	< 0.0001	2.565 (2.320, 2.836)	< 0.0001	2.645 (2.384, 2.935)
48–59	< 0.0001	1.750 (1.551, 2.973)	< 0.0001	1.763 (1.557, 1.997)
Region of residence				
Tigray (ref)	< 0.0001	1	< 0.0001	1
Afar	0.101	1.078 (0.688, 1.689)	0.003	0.683 (0.530, 0.880)
Amhara	0.003	1.398 (1.165, 1.678)	0.113	1.173 (0.963, 1.428
Oromia	0.055	0.890 (0.751, 1.055)	< 0.0001	0.670 (0.538, 0.834
Somali	< 0.0001	0.581 (0.442, .764)	< 0.0001	0.401 (0.312, 0.515
Benishangul-gumuz	0.097	1.161 (0.758, 1.780)	0.429	0.911 (0.723, 1.148)
SNNPR	0.468	0.999 (0.832, 1.199)	0.062	0.800 (0.633, 1.011
Gambela	< 0.0001	0.480 (0.177, 1.306)	< 0.0001	0.467 (0.350, 0.625
Harar	0.004	0.739 (0.282, 1.933)	0.009	0.679 (0.509, 0.908
Addis Ababa	< 0.0001	0.271 (0.179, .409)	< 0.0001	0.476 (0.338, 0.671
Dire dawa	0.945	1.108 (0.562, 2.187)	0.413	0.886 (0.664, 1.183
Mother’s education level				
No education (ref)	< 0.0001	1	< 0.0001	1
Primary	< 0.0001	0.758 (0.685, .840)	0.016	0.870 (0.777, 0.974
Secondary	< 0.0001	0.401 (0.328, 0.491)	< 0.0001	0.606 (0.483, 0.761
Higher	0.0001	0.247 (0.179, .340)	< 0.0001	0.453 (0.319, 0.643
Mother’s BMI				
Overweight (ref)	< 0.0001	1	< 0.001	1
Thin for height	< 0.001	2.167 (1.759, 2.668	< 0.001	2.163 (1.750, 2.673
Normal	< 0.001	1.993 (1.643, 2.418	< 0.001	1.993 (1.638, 2.426
Household wealth index				
Poor (ref)	< 0.001	1	< 0.001	1
Medium	< 0.0001	0.772 (0.693, 0.860)	0.002	0.801 (0.697, 0.921)
Rich	< 0.001	0.555 (0.504.0.612)	< 0.001	0.688 (0.606, 0.781)
Sex of a child				
Female	< 0.001	0.862 (0.790, 0.941)	< 0.001	0.839 (0.765, 0.920)
Size of a child at birth				
Large (ref)	< 0.001	1	< 0.001	1
Medium	0.064	1.098 (0.995, 1.212)	0.003	1.189 (1.062, 1.331
Small	< 0.001	1.475 (1.323, 1.64)	< 0.001	1.509 (1.332, 1.709
Toilet facility				
Have facilities	< 0.001	0.520 (0.442, 0.61)	< 0.001	0.758 (0.642, 0.896
Number of children				
No (ref)	0.003	1	< 0.001	1
1–2	0.523	0.860 (0.543, 1.365	0.013	0.475 (0.264, 0.853
3–4	0.134	0.698 (0.436, 1.117	< 0.001	0.342 (0.188, 0.620
> 4	0.596	0.826 (0.407, 1.677	0.019	0.363 (0.155, 0.849

ref: reference category; COR: crude odds ratio; AOR: adjusted odds ratio; CI: confidence interval

#### Determinants of under-weight

Age of child, sex of a child, mothers’ education level, mothers’ BMI, region, household wealth index, water facility, toilet facility, size of child and a number children were associated with under-weight (P < 0.05). The risk of being underweighted was 1.748, 1.837 times more likely among children that were aged 24–47, and 48–59 months than those aged 0–24 months. Compared to Tigray region, the odds of being under-weighted was 0.741, 0.664 and 0.393 times lower among children from Oromia, Gambella and Addis Ababa respectively.

The risk of being underweight for children whose mother attend primary, secondary and higher education were 0.771, 0.645, and 0.551 times lower than children whose mothers who did not attend formal education. Children from a household with middle and rich economic status were 0.794 and 0.565 times less likely to be under-weighted compared to children living in a household with poor household economic status.

Female children were 0.856 times less likely to be under-weighted as compared to male children. Children who were born with small size were 1.898 times more likely to be under-weighted than children born larger (AOR = 1.898; 95% CL 1.653–2.180) and children who had born with medium size were 1.324 times more likely to be under-weighted than children born larger (AOR = 1.324; 95% CL 1.164–1.507). Children born to underweight mothers (BMI < 18.5) were 3.162 (AOR: 3.162, 95% CI: 2.410, 4.148) times more likely to be underweight compared to Children born to overweight mother (Table 2).

**Table 2:** Significant determinants related to underweight (EDHS, 2016)

Variable	P-value	COR (95% CI)	P-value	AOR (95% CI)
Age of child in months				
0–24	< 0.001	1	< 0.001	1
24–47	< 0.001	1.688 (1.511, 1.886)	< 0.001	1.748 (1.54, 1.965)
48–59	< 0.001	1.706 (1.497, 1.943)	< 0.001	1.837 (1.596, 2.113)
Region of residence				
Tigray (ref)	< 0.001	1	< 0.001	1
Afar	0.010	1.855 (1.159, 2.969)	0.324	1.152 (0.870, 1.524)
Amhara	0.003	1.381 (1.119, 1.703)	0.218	1.149 (0.921, 1.433)
Oromia	0.808	0.976 (0.800, 1.190)	0.020	0.741 (0.575, 0.954)
Somali	0.047	1.338 (1.004, 1.783)	0.481	0.905 (0.686, 1.194)
Benishangul-gumuz	0.011	1.796 (1.143, 2.825)	0.012	1.389 (1.076, 1.793)
SNNPR	0.402	0.912 (0.737, 1.130)	0.010	0.698 (0.530, 0.918)
Gambela	0.590	0.737 (0.242, 2.240)	0.015	0.664 (0.477, 0.925)
Harar	0.826	0.883 (0.290, 2.686)	0.552	0.904 (0.647, 1.262)
Addis Ababa	< 0.001	0.190 (0.101, 0.358)	< 0.001	0.393 (0.231, 0.667)
Dire dawa	0.525	1.278 (0.600, 2.720)	0.647	1.080 (0.778, 1.500)
Mothers’ education level				
No education (ref)	< 0.001	1	< 0.001	1
Primary	< 0.001	0.579 (0.514, 0 .652)	< 0.001	0.771 (0.673, 0.884)
Secondary	< 0.001	0.325 (0.253, 0 .419)	< 0.003	0.645 (0.486, 0.858)
Higher	< 0.001	0.188 (0.122, 0 .288)	< 0.012	0.551 (0.347, 0.876)
Mothers’ BMI				
Overweight (ref)	0.001	1	< 0.001	1
Thin for height	0.001	3.278 (2.504, 4.291)	< 0.001	3.162 (2.410, 4.148)
Normal	0.001	2.309 (1.786, 2.985)	< 0.001	2.245 (1.733, 2.908)
Household wealth index				
Poor (ref)	< 0.001	1	< 0.001	1
Medium	< 0.001	0.735 (0.650, 0.832)	0.003	0.794 (0.681, 0.927)
Rich	< 0.001	0.473 (0.421, 0 .531)	< 0.001	0.565 (0.487, 0.654)
Sex of a child				
Female	0.013	0.885 (0.803, 0.975)	0.003	0.856 (0.773, 0.948)
Size of a child at birth	< 0.001	1		
Large (ref)	< 0.001	1.286 (1.141, 1.449)	< 0.001	1
Medium	< 0.001	2.085 (1.839, 2.364)	< 0.001	1.324 (1.164, 1.507)
Small			< 0.001	1.898 (1.653, 2.180)
Water facility	0.034	1.117 (1.008, 1.238)		
Safe	< 0.001	0.562 (0.462, 0.484)	0.012	1.151 (1.031, 1.284)
Toilet facility				
Have facility	< 0.001	0.403 (0.342, 0.474)	0.005	0.757 (0.623, 0.920)

#### Determinants of wasting

Results of multivariable binary logistic regression model showed that the age of a child, sex of a child, mothers’ education level, household wealth index, a region of residence, water facility, and family size were significantly associated with wasting. Children of the rich household were less likely to be wasted compared to children living in a household with poor household economic status. The risk of being wasted was 0.52 and 0.607 times lower among children of 25–47 and 48–59 months than those 0–24 months. Compared to children from the Tigray region, the odds of being wasted of children from Somali region was 1.671 times higher. The odds of being wasted in SNNPR and Addis Ababa were 0.365 and 0.338 times lower compared to Tigray region respectively. The odds of being wasted was 0.778 times lower among female children than male children (AOR = 0.778, 95% CI 0.681, 0.889). The odds of being wasted was 1.223 times higher among children who lived in household members of 6–10 than children who had lived in household members of 1–5 (AOR = 1.223, 95% CI 1.066, 1.403) (Additional file 2).

#### Associations between children’s anemia and malnutrition

This study showed that among stunted, underweighted, and wasted children, 61%, 64.3%, and 68.2% were anemic respectively. Moreover, the percentages of stunting, wasting, and underweighting were higher among anemic children as compared to no-anemic children. Stunted children were 1.222 times more likely to be anemic compared to those of not stunted (AOR: 1.222, 95% CI 1.101, 1.356). Underweighted children were 1.222 times more likely to be anemic compared to those of not underweight (AOR: 1.222, 95% CI 1.077, 1.386). Wasted children were 1.557 times more likely to be anemic compared to those of not wasted (AOR: 1.557, 95% CI 1.315, 1.844) (Table 3).

**Table 3:** Association of malnutrition with anemia among under-five children in Ethiopia

Variables	Category	Not anemic	Anemic (%)	P-value	AOR (95% CI)
Stunting	Not stunted	2207 (45.7)	2619 (54.3)	–	1
	Stunted	1347 (39.0)	2106 (61.0)	< 0.001	1.222 (1.101, 1.356)
Underweight	Not underweight	2798 (45.4)	3371 (54.6)	–	1
	Underweight	751 (35.7)	1354 (64.3)	0.002	1.222 (1.077, 1.386)
Wasting	Not wasted	3295 (44.1)	4183 (55.9)	–	1
	Wasted	253 (31.8)	543 (68.2)	< 0.001	1.557 (1.315, 1.844)

AOR: adjusted odds ratio; CI: confidence interval

### Discussion

In this study, the prevalence of malnutrition and associated factors in Ethiopia was assessed. The prevalence of stunting, underweighting, and wasting were 38.3%, 23.3%, and 10.1% respectively. These prevalence were relatively lower than the previous study conducted in Ethiopia [9, 10] and in Tanzania [5], but it was higher than the study conducted in Nairobi, Kenya [11].

In this study, as the age of a child increase, the probability of a child to be stunted and underweighted will be increased. This finding was in line with the studies that conducted in Ethiopia, in which poor nutritional status of children was associated with the old age of children [12–14]. In all the three forms of malnutrition (stunting, underweight and wasting), the risk of malnutrition was less prevalent among females than males. This finding was consistent with previous findings [15–17]. This study revealed that the levels of malnutrition had a significant regional variation ranging from 14.6% in Addis Ababa to over 46.7% in Amhara regions of the country. This finding is similar with [10, 18].

Children whose mothers had primary and above educational level were significantly less likely to be stunted and underweighted as compared to children whose mothers had never attended formal education. This finding was consistent with the study conducted in Ethiopia [10] and Bangladesh [19] which showed that as mothers’ educational level increase, the likelihood of the children to be stunted and underweighted will be decreased. Mothers with BMI less than 18.5 (underweight) were more likely to have stunted, underweighted and wasted children as compared to overweighted mothers. This finding is similar with other previously conducted studies [10, 19, 20].

As of this study, children who were smaller at birth were more likely to be stunted and underweighted. This finding was supported by study conducted previously in SNNPR, Ethiopia. [13]. A similar study that conducted in Dale Woreda, Southern Ethiopia showed that the larger the family size, the poorer nutritional status of children would be resulted [17]. In the current study, anemia and malnutrition of children were highly associated with that anemic children were more likely to be malnutrition as compared to non-anemic [7].

## Conclusions

The prevalence of stunting was still high in Ethiopia. The key determinants of malnutrition in Ethiopia were the child age, maternal education, region, household wealth status, religion, sex of child, number of children, a child size, water and toilet facility. The influence of these factors should be considered to develop strategies for reducing malnutrition in Ethiopia. Finally, improving living standards of children is important to get a better health care, reduces child malnutrition, and child mortality.

## Limitations of the study

This study was based on cross-sectional study design. Thus, the authors did not see the seasonal variation of malnutrition status and establish causal relationship. There were some missing values for some variables in the dataset. Therefore, the authors fail to consider some important factors which could affect the interpretation of the results.

## Additional files

**Additional file 1.** Characteristics of the Study Participants (EDHS, 2016). Descriptive statistics of study variables.

**Additional file 2.** Results of multivariable logistic regression to identify the significant determinants related to wasting.

## Data Availability

The datasets used and analyzed during the current study are available from the corresponding author on reasonable request (in SPSS code).

## Abbreviations

SPSS: Statistical Package for Social Science
COR: crude odds ratio
AOR: adjusted odds ratio
EDHS: Ethiopian Demographic and Health Survey
CSA: Central Statistical Agency
BMI: body mass index
WHO: World Health Organization
SNNPR: Southern Nations, Nationalities and People Region
