# Supplementary material for: Prevalence of malnutrition and associated factors among under-five children in Ethiopia: evidence from the 2016 Ethiopia Demographic and Health Survey
Source: BMC Res Notes. 2019 Jul 11;12:391. doi: 10.1186/s13104-019-4444-4 (PMC6624874; doi:10.1186/s13104-019-4444-4)
Supplement: Supplementary file 1 — Additional file 1. Characteristics of the Study Participants (EDHS, 2016). Descriptive statistics of study variables. [file 13104_2019_4444_MOESM1_ESM.docx]

**Additional file 1** Characteristics of the Study Participants (EDHS, 2016).

| Variables | Category | Frequency | Percentage |
| --- | --- | --- | --- |
| Age of a child in months | 0-24 | 4147 | 43.7 |
|  | 25-47 | 3413 | 35.9 |
|  | 48-59 | 1935 | 20.4 |
| Region of Residence | Tigray | 647 | 6.8 |
|  | Afar | 90 | 0.9 |
|  | Amhara | 1865 | 19.6 |
|  | Oromia | 4164 | 43.9 |
|  | Somali | 393 | 4.1 |
|  | Benishangul | 100 | 1.1 |
|  | SNNPR | 1947 | 20.5 |
|  | Gambela | 22 | 0.2 |
|  | Harari | 20 | 0.2 |
|  | Addis Adaba | 210 | 2.2 |
|  | Dire Dawa | 36 | 0.4 |
| Mothers’ education level | No education | 6225 | 65.6 |
|  | Primary | 2600 | 27.4 |
|  | Secondary | 453 | 4.8 |
|  | Higher | 217 | 2.3 |
| Mother’s BMI | Thin for height | 1874 | 20.1 |
|  | Normal | 6877 | 73.6 |
|  | Overweight | 593 | 6.3 |
| Household wealth index | Poor | 4414 | 46.5 |
|  | Middle | 2001 | 21.1 |
|  | Rich | 3079 | 32.4 |
| Sex of a child | Male | 4851 | 51.1 |
|  | Female | 4644 | 48.9 |
| Residence area | Urban | 1046 | 11.0 |
|  | Rural | 8449 | 89.0 |
| Size of child at birth | large | 2980 | 31.4 |
|  | Medium | 4004 | 42.2 |
|  | Smaller | 2510 | 26.4 |
| Water facility | Unsafe | 6058 | 63.8 |
|  | Safe | 3437 | 36.2 |
| Toilet type | No facilities | 8661 | 91.2 |
|  | Have facilities | 833 | 8.8 |
| Number of children | No child | 75 | 0.8 |
|  | 1-2 | 7750 | 81.6 |
|  | 3-4 | 1614 | 17.0 |
|  | >4 | 56 | 0.6 |
| Family size | 1-5 | 4088 | 43.1 |
|  | 6-10 | 5131 | 54.0 |
|  | >10 | 275 | 2.9 |
