# Supplementary material for: Prevalence of malnutrition and associated factors among under-five children in Ethiopia: evidence from the 2016 Ethiopia Demographic and Health Survey
Source: BMC Res Notes. 2019 Jul 11;12:391. doi: 10.1186/s13104-019-4444-4 (PMC6624874; doi:10.1186/s13104-019-4444-4)
Supplement: Supplementary file 2 — Additional file 2. Results of multivariable logistic regression to identify the significant determinants related to wasting. [file 13104_2019_4444_MOESM2_ESM.docx]

**Additional file 2** Significant Determinants Related to Wasting (EDHS, 2016)

| **Variables** | P-value | COR(95%CI) | P-Value | AOR(95% CI) |
| --- | --- | --- | --- | --- |
| **Age of a child**  **in month** |  |  |  |  |
| 0-24(ref) | 0.000 | 1 | 0.000 | 1 |
| 25-47 | 0.000 | 0.529(.453, .617) | 0.000 | 0.520(0.447,0.606) |
| 48-59 | 0.000 | 0.467(.383, .569) | 0.000 | 0.607(0.506,0.728) |
| **Region of residence** |  |  |  |  |
| Tigray(ref) | 0.000 | 1 | 0.000 | 1 |
| Afar | 0.000 | 1.855(1.421, 2.421) | 0.241 | 1.246(0.862,1.800) |
| Amhara | 0.266 | 0.843(.624, 1.139) | 0.200 | 0.819(0.602,1.112) |
| Oromia | 0.528 | 0.918(.704, 1.198) | 0.068 | 0.727(0.517,1.024) |
| Somali | 0.000 | 2.075(1.621, 2.658) | 0.004 | 1.671(1.173,2.380) |
| Benishangul-gumuz | 0.462 | 0.889(.649, 1.217) | 0.152 | 0.765(0.530,1.104) |
| SNNPR | 0.000 | 0.519(.378, .714) | 0.000 | 0.365(0.243,0.549) |
| Gambela | 0.100 | 1.300(.951, 1.777) | 0.439 | 0.847(0.557,1.289) |
| Harar | 0.911 | 0.980(.688, 1.397) | 0.708 | 0.921(0.597,1.419) |
| Addis Ababa | 0.000 | 0.263(.146, .475) | 0.000 | 0.338(0.183,0.622) |
| Dire dawa | 0.398 | 0.849(.580, 1.242) | 0.198 | 0.744(0.475,1.167) |
| **Mothers’ BMI** |  |  |  |  |
| Overweight(ref) | 0.000 | 1 | 0.000 | 1 |
| Thin for height | 0.000 | 2.942(1.999,4.330) | 0.000 | 2.899(1.964,4.277) |
| Normal | 0.000 | 1.938(1.336, 2.813) | 0.001 | 1.915(1.316,2.787) |
| **Household**  **wealth index** |  |  |  |  |
| Poor(ref) | 0.000 | 1 | 0.000 | 1 |
| Medium | 0.108 | 0.870(0.734,1.031) | 0.375 | 0.910(0.739,1.121) |
| Rich | 0.000 | 0.589(0.501, .694) | 0.000 | 0.671(0.564, 0.798) |
| **Sex of a child** |  |  |  |  |
| Female | 0.001 | 0.809(0.711,0.921) | 0.000 | 0.778(0.681, 0.889) |
| **Water facility** |  |  |  |  |
| Safe | 0.028 | 1.163(1.016,1.331) | 0.014 | 1.194(1.037, 1.375) |
| **Family Size** |  |  |  |  |
| 1-5(ref) | 0.002 | 1 | 0.014 | 1 |
| 6-10 | 0.001 | 1.266(1.101, 1.454) | 0.004 | 1.223(1.066, 1.403) |
| Above 10 | 0.036 | 1.489(1.027, 2.159) | 0.978 | 1.005(0.683, 1.480) |
